# Programmable gene regulation for metabolic engineering using decoy transcription factor binding sites

**DOI:** 10.1093/nar/gkaa1234

**Published:** 2020-12-24

**Authors:** Tiebin Wang, Nathan Tague, Stephen A Whelan, Mary J Dunlop

**Affiliations:** Molecular Biology, Cell Biology & Biochemistry, Boston University, Boston, MA 02215, USA; Biological Design Center, Boston University, Boston, MA 02215, USA; Biological Design Center, Boston University, Boston, MA 02215, USA; Biomedical Engineering, Boston University, Boston, MA 02215, USA; Chemistry, Boston University, Boston, MA 02215, USA; Molecular Biology, Cell Biology & Biochemistry, Boston University, Boston, MA 02215, USA; Biological Design Center, Boston University, Boston, MA 02215, USA; Biomedical Engineering, Boston University, Boston, MA 02215, USA

## Abstract

Transcription factor decoy binding sites are short DNA sequences that can titrate a transcription factor away from its natural binding site, therefore regulating gene expression. In this study, we harness synthetic transcription factor decoy systems to regulate gene expression for metabolic pathways in *Escherichia coli*. We show that transcription factor decoys can effectively regulate expression of native and heterologous genes. Tunability of the decoy can be engineered via changes in copy number or modifications to the DNA decoy site sequence. Using arginine biosynthesis as a showcase, we observed a 16-fold increase in arginine production when we introduced the decoy system to steer metabolic flux towards increased arginine biosynthesis, with negligible growth differences compared to the wild type strain. The decoy-based production strain retains high genetic integrity; in contrast to a gene knock-out approach where mutations were common, we detected no mutations in the production system using the decoy-based strain. We further show that transcription factor decoys are amenable to multiplexed library screening by demonstrating enhanced tolerance to pinene with a combinatorial decoy library. Our study shows that transcription factor decoy binding sites are a powerful and compact tool for metabolic engineering.

## INTRODUCTION

Metabolic flux in microbes is coordinated by transcription factors that dictate gene expression levels for genes encoding enzymes that carry out necessary chemical conversions. Removing the effect of transcription factors can alter expression of genes and redirect native metabolic pathways (1,2). Strategies for doing this can generally be divided into two groups: complete removal and partial removal of the transcription factor. For the first, traditional gene knock-out strategies can be used to remove the gene encoding the transcription factor. This approach has been shown to enhance microbial tolerance towards biofuels (3–6) and increase production of amino acids (7–11). Although techniques for generating gene knock-out are well-established, genome editing is a labor intensive process and can be difficult to multiplex. In addition, since transcription factors can play broad physiological roles, completely removing the gene is frequently associated with detrimental effects, such as fitness costs (7,12). For example, knocking out *argR* in *Escherichia coli* can substantially increase the production of arginine, but reduces cell growth by 50% (7). This growth deficit inevitably leads to selective pressure against engineered cells, potentially reducing genetic stability, which is an important consideration during the scale up process (13–17).

Partial removal strategies include knock-down approaches such as using CRISPRi or sRNA to downregulate gene expression (7,10,18). These approaches are straightforward to design and can be used to achieve multiplexed regulation. Importantly, partially removing the effect of a transcription factor can redirect metabolic flux at intermediate levels that are better tolerated by the cell (7,10). For example, compared to the 50% growth deficit resulting from an *argR* knock-out, knock-down of *argR* with CRISPRi in *E. coli* reduced growth by 30% while maintaining similar arginine productivity (7). Such partial removal strategies have been employed to increase yields for various target products, such as the nylon precursor cadaverine in addition to the amino acid arginine (7,10). However, CRISPRi can show significant toxicity and off-target activity (19,20). In addition, the size of knock-down systems is large and may place a practical limit on the number of genes that can be introduced into the host cell. For example, CRISPRi knock-down requires dCas9 and an sgRNA, accounting for approximately 5 kilobases of additional genetic material. As a result, it remains challenging to control transcription factor levels using existing approaches. The ideal strategy would be straightforward to design and construct, have low fitness cost, and exhibit high compatibility with the large heterologous pathways involved in metabolic engineering.

Decoy binding sites provide a potential platform for regulation of transcription factor activity. Transcription factor decoys are short DNA sequences (∼30 bp) that can act like a sponge to soak up free transcription factors in the cell, titrating them away from their functional promoters to alter gene expression. Natural examples of this strategy exist, where decoy binding sites sequester transcription factors to regulate gene expression without relying on further transcription or translation (21,22). Therapeutically, decoy sequences can serve as an avenue for gene therapy (23–25). In this approach, decoy sequences are delivered to the body to alter disease through transcriptional changes. Transcription factor decoys have also been incorporated into synthetic biology designs, but have been limited to synthetic gene circuits to either alter dynamics or lower noise (26–28). Another recent example from Wang *et al.* (29) demonstrates the use of decoys to activate silent biosynthetic gene clusters. However, the systematic application of transcription factor decoys to the regulation of endogenous genes and metabolic pathways remains largely unexplored. Here, we seek to demonstrate the utility of transcription factor decoys for metabolic engineering.

We designed and engineered decoys that allow multiplexed and tunable regulation of transcription factors related to metabolism. We show that the synthetic decoy systems can effectively alter transcriptional outputs from promoters in the regulon of a targeted transcription factor. Importantly, the decoy effect is tunable, which we show by changing plasmid copy number and also the sequence of the decoy. As a metabolic engineering application, we use arginine biosynthesis as a showcase and design synthetic decoy systems for the arginine production pathway repressor ArgR. Our results show that metabolic flux to the arginine production pathway is increased by the introduction of a synthetic decoy site. Using liquid chromatography–mass spectrometry (LC–MS), we demonstrate that production is increased 16-fold compared to the parental production strain without a detectable impact on growth. Further, we demonstrate that the decoy-based production strain retains higher genetic integrity than a similar system using an *argR* knock-out. After a cyclic culturing process to simulate the potential for genetic drift during scale-up, we found that 87% of the knock-out strains obtained mutations, while no mutations were detected in the decoy-based production strain. Lastly, we establish the use of decoy libraries for phenotypic screening. Using a combinatorial library of decoy binding sites, we identify several single and double decoys containing binding sites for regulators of tolerance genes that significantly increase pinene tolerance. These results indicate that transcription factor decoys have excellent potential as a tool for metabolic engineers due to their low fitness cost and compact size.

## MATERIALS AND METHODS

### Strains

We used *E. coli* BW25113 as the wild type strain. Δ*argR* was derived from *E. coli* BW25113 and we deleted the *argR* gene using homologous recombination (30). We used the forward primer 5′-AAG CAA GAA GAA CTA GTT AAA GCA TTT AAA GCA TTA CTT AAA GAA GAG AAg tgt agg ctg gag ctg ctt c-3′ and the reverse primer 5′-CCT GGT CGA ACA GCT CTA AAA TCG CTT CGT ACA GGT CTT TGA CTG TGA AAa ttc cgg gga tcc gtc gac c-3′. Capitalized letters indicate the homologous recombination extension.

The ArgA* production strain was created by transforming *E. coli* BW25113 with an ArgA* production plasmid containing P_argA_-*argA* (H15Y). To construct the ArgA* production plasmid, we amplified the promoter and gene region of *argA*, P_argA_-*argA*, from *E. coli* BW25113 using the forward primer 5′- GCC TCT CCC GAG CAA AAG -3′ and reverse primer 5′- TTA CCC TAA ATC CGC CAT CAA C -3′. We then introduced mutations in the PCR product of P_argA_-*argA* to create P_argA_-*argA* (H15Y) using the forward primer 5′- GGG ATT CCG CTA TTC AGT TCC -3′ and reverse primer 5′-TTA CCC TAA ATC CGC CAT CAA C-3′. P_argA_-*argA* (H15Y) was then cloned on the low-copy (SC101) plasmid pBbS5C from Lee *et al.* (31).

### Transcription factor decoy plasmid construction

#### LacI decoy plasmid

We cloned the LacI binding site sequence AATTGTGAGCGGATAACAATT into the p15A replication origin plasmid pBbA5A, or ColE1 replication origin plasmid pBbE5A, using the forward primer 5′-AAT TGT GAG CGG ATA ACA ATT cca tcg ttg aac agt acg aac-3′, and reverse primer 5′-AAT TGT TAT CCG CTC ACA ATT cca tca aac agg att ttc gcc-3′. For cloning into the pMB1 (high copy derivative) plasmid pUC19, we used the forward primer 5′-AAT TGT GAG CGG ATA ACA ATT taa tgc agc tgg cac gac-3′, and reverse primer 5′-AAT TGT TAT CCG CTC ACA ATT ggt ttg cgt att ggg cgc-3′. Capitalized letters indicate the homologous region. All plasmids were derived from the BglBrick library described in Lee *et al.* (31).

#### TetR decoy plasmid

We used the forward primer 5′-TCC CTA TCA GTG ATA GAG Ata atg cag ctg gca cga c-3′, and reverse primer 5′-TCT CTA TCA CTG ATA GGG Agg ttt gcg tat tgg gcg c-3′ to clone the TetR binding site sequence TCCCTATCAGTGATAGAGA into the pMB1 (high copy derivative) plasmid pUC19.

#### ArgR consensus decoy plasmid

For the ArgR consensus decoy plasmid, we introduced the consensus ArgR binding site sequence TTATTTGCATAAAAATTCATT into the ColE1 replication origin plasmid pBbE5A using the forward primer 5′-TTA TTT GCA TAA AAA TTC ATT TGT ATG CAC Agc tga agg tcg tca ctc ca-3′, and reverse primer 5′-AAT GAA TTT TTA TGC AAA TAA CAG TCA GCC CCc cac cgt ctt tca gtt tca ga-3′.

#### ArgR decoy library plasmid

For the ArgR decoy library plasmid, we introduced the ArgR binding site sequence with randomized sequence WWWWWTGMATRAWWATTCABT (W:A,T; M:A,C; R:A,G; B:C,G,T) into the ColE1 replication origin plasmid pBbE5A. The position weight matrix was calculated using the 31 ArgR binding sites listed in Santos-Zavaleta *et al.* (32) using the method described in Hertz *et al.* (33).

#### ArgR decoy inducible copy number plasmid

For the ArgR consensus decoy plasmid, we introduced the consensus ArgR binding site sequence TTATTTGCATAAAAATTCATT into a plasmid with an IPTG-inducible phage P1 replication system with tunable copy number (34).

### LacI and TetR decoy test

Bacteria were cultured in M9 minimal medium with 5 g/l glucose at 37°C with 200 rpm shaking. Overnight cultures inoculated from a single colony were diluted 1:50 in M9 minimal medium with 5 g/l glucose and selective antibiotics for plasmid maintenance, where required. The diluted cultures were then precultured for 3 h, 1 mM IPTG or 100 nM aTc was added (where indicated), then grown at 37°C with 200 rpm shaking. Red fluorescent protein (RFP) readings (excitation 580 nm, emission 610 nm) were taken using a BioTek Synergy H1m plate reader (BioTek, Winooski, VT) after 18 h of incubation at 37°C with 200 rpm shaking. Negative and positive control experiments used strains with only the reporter plasmid present (negative = no induction with IPTG/aTc, positive = induced with IPTG/aTc).

### ArgR decoy test

Bacteria were cultured in M9 minimal medium with 5 g/l glucose at 37°C with 200 rpm shaking. Overnight cultures inoculated from a single colony were diluted 1:50 in M9 minimal medium with 5 g/l glucose and selective antibiotics for plasmid maintenance, where required. The diluted cultures were then precultured for 2 h, arginine and IPTG were added when needed at the concentrations indicated in figure captions, then cultures were grown at 37°C with 200 rpm shaking. Green fluorescent protein (GFP) readings (excitation 480 nm, emission 510 nm) were taken using a BioTek Synergy H1m plate reader after 4 h of incubation with shaking at 37°C.

Normalized fluorescence was calculated with the following equation:

$$\begin{eqnarray*} {\rm Normalized}\ {\rm fluorescence} = \frac{{\frac{{{\rm GFP} ( {{\rm{exp}}} )}}{{{\rm OD660} ( {{\rm{exp}}} )}}}}{{\frac{{{\rm GFP} ( {{\rm ctl}} )}}{{{\rm OD660} ( {{\rm ctl}} )}}}}, \end{eqnarray*}$$

where GFP (exp) and OD_660_ (exp) are GFP and optical density readings of arginine pathway reporters in the strain with or without the ArgR decoy and with or without arginine treatment. GFP (ctl) and OD_660_ (ctl) are GFP and optical density reading for the strain harboring the arginine pathway reporters without arginine treatment. Strains containing the pathway reporters measured at 0, 0.06, 0.6 and 6 mM arginine contain only the reporter plasmid and not the plasmid containing the decoy.

### Arginine production experiments

Overnight cultures of the production strains were diluted 1:50 in 5 ml M9 minimal medium with 5 g/l glucose with antibiotics, when appropriate. Diluted cultures were then grown for 24 h at 37°C with 200 rpm shaking. Bacterial cultures were then placed on ice and lysed with 10 cycles of sonication (10 s ON, 30 s OFF, 20% amplitude). 40 μl of cell lysate was mixed with acetone at a 1:8 ratio, vortexed and kept on ice for 30 min. Samples were then centrifuged at 15 000 rcf for 10 min at 10°C to pellet proteins and lipids. Supernatant was transferred to a new tube, leaving behind the protein pellet. The sample was then dried in a speed vacuum centrifuge and then reconstituted by adding 40 μl H_2_O with 0.1% formic acid and vortexed. Samples were placed on ice for 15 min and centrifuged to remove any residual protein or lipid. Supernatant was then transferred to a glass LC vial with a glass insert and measured using an Agilent HPLC 1100 series auto sampler.

### LC–MS/MS

An Agilent HPLC 1100 series was used with a Phenomenex Kinetex 2.6 μm F5 100 Å 150 mm × 2.1 mm column (PN 00F-4723-AN). Arginine was analyzed on a Sciex API 4000 mass spectrometer triple quadrupole in positive polarity with a targeted Q1 Mass of 175 100 Da and a Q3 mass of 70 000 Da, with a dwell time 20.0 ms, declustering potential (DP) 50.0 V, entrance potential (EP) 10.0 V, collision energy (CE) 32.0 V, and a collision cell exit potential (CXP) 9.0 V. Sample was injected at l μl onto the column and was analyzed at a gradient of 97% H_2_O and 0.1% formic acid (Buffer A) and 3% acetonitrile and 0.1% formic acid (Buffer B) at 0–2.0 min, 3% Buffer B at 2.0 min to 95% Buffer B at 7 min, 95% Buffer B to 8.0 min, to 3.0% Buffer B at 8.5–10.0 min at a flow rate of 200 μl/min. Sciex MultiQuant 3.0.3 software was used to analyze data and calculate concentrations from a linear plot of arginine standards.

### ArgA* genetic integrity test

ArgA*/Δ*argR* or ArgA*/decoy strains were cultured in M9 minimal medium with 5 g/l glucose at 37°C with 200 rpm shaking. Overnight cultures inoculated from a single colony were diluted 1:50 in M9 minimal medium with 5 g/l glucose and antibiotics for plasmid maintenance and cultured for 24 h. We refer to this as cycle 1. Every 24 h, we diluted the culture 1:100 in fresh M9 minimal medium with 5 g/l glucose and antibiotics and repeated this procedure until cycle 6.

Before dilution each cycle, we isolated DNA to sequence regions of interest. The target regions of interest for sequencing were the coding sequence for argA* for the Δ*argR* production strain, and argA* and the decoy region in the decoy-based production strain. We isolated plasmids from the bacterial cultures using the GenCatch™ Plasmid DNA Mini-Prep Kit. Isolated plasmids were then amplified with PCR with the following primers and sequenced using the forward PCR primers. For the ArgA* plasmid we used forward primer 5′-GCC TCT CCC GAG CAA AAG-3′ and reverse primer 5′-TAT AAA CGC AGA AAG GCC CAC-3′. For the decoy plasmid, we used forward primer 5′-CTG CGT GGT ACC AAC TTC C-3′ and reverse primer 5′-CCG AAC GCC CTA GGT ATA AAC-3′.

### One-pot decoy library

To construct single and double decoy libraries, forward and reverse oligos containing each transcription factor decoy site with 5′ overhangs were annealed by heating the oligo mix (1 μM) to 95°C and allowing the heat block to return to room temperature. 1 pmol of the oligo mix was added to a 20 μl Golden Gate reaction containing 100 ng destination plasmid (pATT-DEST, Addgene #79770), 10 units BsaI (NEB #R3733), 10 units PNK (Thermo Fisher), and 1 unit T4 ligase with associated buffer (Promega). The following thermocycler program was run: 25 cycles of 37°C for 2 min and 16°C for 5 min, followed by 60°C for 10 min (final digestion) and 80°C for 10 min (heat inactivation). Successful decoys replace *lacZ* on the plasmid. Single decoys were designed using oligo overhangs that match the destination plasmid. Double decoy constructs were designed such that the reverse oligo of the first decoy and the forward oligo of the second decoy match and are distinct from the destination plasmid.

### Pinene tolerance screen

Single and double decoy libraries were created to screen for pinene tolerance. Libraries for single and double decoys were created in separate Golden Gate reactions and mixed prior to transformation. The transformation was recovered for 1 h at 37°C prior to addition of antibiotic. At this point, a small subset of the recovery culture was plated on LB agar plates containing antibiotic to ensure adequate transformation efficiency for library coverage. The culture was grown for an additional 4 h before adding 0.5% α-pinene (v/v) in 5 ml LB medium. The cultures were then allowed to grow for 36 h at 37°C with 200 rpm shaking. A 10^−6^ dilution of the final culture was plated. We conducted six replicate selection experiments. In each replicate, eight individual colonies of the library pre- and post-selection were sequenced to reveal the decoy binding sites present. Pre-selection sequencing revealed a population of diverse single and double decoys. The selection protocol, starting from the initial transformation, was repeated in parallel in six biological replicates, with five of six experiments resulting is cell growth. All five selection experiments converged on a single decoy plasmid present, meaning all eight sequenced colonies were identical. To validate decoy binding sites revealed by the screen, individual plasmids were isolated and re-transformed into fresh cells to rule out the possibility of genomic mutations. Colonies were grown overnight and diluted 1:200 into 24-well plates with LB medium containing 0.5% α-pinene (v/v). Cells with a plasmid of the same backbone, but without a decoy, were grown in parallel as a negative control. The OD_600_ was monitored to quantify cell growth.

## RESULTS

### Altering gene expression with the decoy system

**Figure 1.**
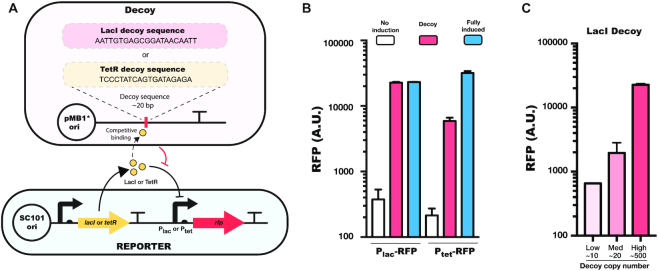
Decoy system for the transcriptional repressors LacI and TetR. (**A**) Schematic view of the decoy plasmid design for LacI and TetR. The LacI or TetR decoy sequence is inserted into a plasmid, where the sequence is immediately followed by a transcription terminator. The copy number of this plasmid depends on the experiment, but pMB1* (∼500 copies) is shown as an example. The reporter plasmid contains the P_lac_ or P_tet_ promoter driving expression of the gene for red fluorescent protein (*rfp*) on a SC101 origin plasmid (∼5 copies). (**B**) P_lac_ or P_tet_ promoter expression levels at 18 h with no induction (no decoy plasmid and no inducer), with decoy (decoy plasmid and no inducer), and fully induced (no decoy plasmid and either 1 mM IPTG or 100 nM aTc). (**C**) Regulatory effects of the LacI decoy system in low copy (p15A, ∼10 copies), medium copy (ColE1, ∼20 copies), or high copy (pMB1*, ∼500 copies) origin of replication plasmids. Error bars show standard errors from *n* = 3 biological replicates.

To investigate the effects of transcription factor decoys, we first employed two widely used inducible promoters, P_lac_ and P_tet_, which are controlled by the transcription factors LacI and TetR, respectively. To construct a decoy system for each, we inserted a LacI or TetR binding site sequence acquired from the P_lac_ or P_tet_ promoter region into a high copy plasmid. To avoid unexpected transcription from insertion of the decoy binding site, we placed a transcription terminator immediately downstream of the decoy sequence (Figure 1a).

First, to test the effects of the LacI and TetR decoys, we co-transformed the decoy system with a reporter plasmid consisting of either P_lac_ or P_tet_ driving expression of the gene for red fluorescent protein (*rfp*) (Figure 1A). We found that the decoys can effectively activate *rfp* expression by relieving repression of the promoter. The LacI decoy allowed the P_lac_ promoter to be expressed at a level comparable to saturating levels of IPTG induction (Figure 1B, Supplementary Figure S1). The TetR decoy results were similar, with significantly elevated expression from P_tet_, though in this case the decoy system achieved expression levels that were not quite as high as in the fully induced conditions (Figure 1B).

To test whether the titration effect is a function of the copy number of the decoy, we introduced the LacI decoy into plasmids with different replication origins: p15A, ColE1, and pMB1*, representing low (∼10), medium (∼20) and high (∼500) copy numbers plasmids. Using the P_lac_-RFP reporter, we observed a clear trend of increased transcriptional activity as the copy number of the decoy plasmid is increased (Figure 1C). This suggests that the transcriptional effect can be tuned by controlling copy numbers in the cell. These results also suggest that tens to hundreds of copies of the decoy site are necessary to titrate the transcription factor in this case. For alternative transcription factor and decoy site pairs where fewer decoy sites are sufficient to alter expression, an alternative strategy where multiple decoy sites are added in tandem could play a similar role.

### Controlling a native metabolic pathway using the synthetic decoy system

**Figure 2.**
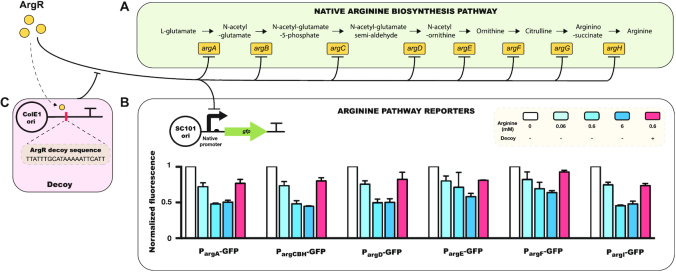
ArgR decoy for regulating the native arginine biosynthesis pathway. (**A**) Schematic view of the native arginine production pathway in *E. coli*. (**B**) Up-regulation of arginine production pathway genes by introduction of the ArgR decoy system in a plasmid with ColE1 origin. Expression of reporters are normalized to the values from the 0 mM arginine case. Error bars show standard error from *n* = 3 biological replicates. (**C**) Design of the ArgR decoy system.

We next sought to test whether the decoy effect could be used to control the activity of a native metabolic pathway. To do this, we focused on arginine biosynthesis as a model system. The arginine production pathway is regulated by the transcription factor ArgR, which controls over 30 targets (32,35) and functions as a strong repressor in the presence of arginine (36), preventing its overproduction (Figure 2A).

To establish a baseline for the minimum and maximum levels of ArgR-regulated promoter activities, we conducted tests with and without arginine on native promoters driving green fluorescent protein (*gfp*) expression (Figure 2b). We employed a set of six transcriptional reporters for the arginine production pathway: P_argA_-GFP, P_argCBH_-GFP, P_argD_-GFP, P_argE_-GFP, P_argF_-GFP and P_argI_-GFP. We measured the transcriptional activity of all six reporters upon supplementation with 0.06 mM, 0.6 mM, or 6 mM arginine. In the presence of arginine, ArgR binds arginine causing repression of genes in the pathway. As expected, increasing concentrations of arginine have a clear negative impact on the transcriptional activity of all reporters (Figure 2B).

We next constructed a decoy system for ArgR to test whether we could use it to restore transcriptional activity of the production pathway, even in the presence of arginine. To test this, we designed an ArgR decoy, which consists of a 21 bp artificial consensus sequence (Figure 2C). To determine the consensus, we calculated the strict consensus region of all 31 known ArgR binding sites based on its position weight matrix (33). We co-transformed the decoy system with each of the arginine pathway reporters and measured the transcriptional activity in the presence of 0.6 mM arginine. We chose 0.6 mM arginine because it is a production-relevant concentration (7) and most arginine pathway genes reach a repression plateau above this concentration (Figure 2B). In the strain containing the ArgR decoy with arginine present, we observed a notable restoration of transcriptional activity with all six reporters, suggesting that the decoy system can effectively titrate ArgR away from its genomic targets and up-regulate the arginine production pathway (Figure 2B). It is notable that the ArgR decoy of ∼20 copies can achieve transcriptional restoration considering there are >30 binding sites within the genome. We speculate that this could result from preferential occupancy of the consensus binding sequence.

### Tunable regulation of the transcription factor decoy

**Figure 3.**
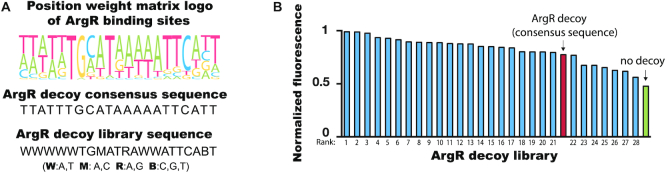
Tunable control of the ArgR decoy system. (**A**) Sequences in the ArgR decoy library. (**B**) Regulatory effects of different sequences in the ArgR decoy library in 0.6 mM arginine. Library members are sorted from high (most effective as a decoy) to low (least effective). Exact sequences are provided for each decoy variant in Supplementary Table S1.

To evaluate whether the decoy effect can be tuned by modifying the sequence of the ArgR binding site, we constructed a library of ArgR sites (Figure 3A) and tested the decoy effect using a P_argA_-GFP reporter. We found that although all members in the ArgR decoy library share the same position weight matrix logo, sequence differences in the non-conservative region of the ArgR binding sites resulted in variants with a range of impacts in 0.6 mM arginine treatment, from no effect to full restoration of transcriptional activity, suggesting that the decoy strength is highly dependent on the DNA sequence and is tunable if the binding sequence is modified (Figure 3B).

Because the decoy effect is a function of its copy number (Figure 1C), we reasoned that an alternative way to engineering tunability would be to vary the plasmid copy number. To achieve this, we employed a plasmid with an IPTG-inducible phage P1 replication system for tunable copy number (34) and incorporated the ArgR consensus decoy site into the inducible copy number plasmid (Supplementary Figure S2a). We measured transcriptional activity of an arginine pathway reporter (P_argA_-GFP) in the presence of 0.6 mM arginine. As expected, we observed a trend of increased expression of the arginine pathway reporter with increased IPTG induction, suggesting that the decoy effect can be controlled by fine-tuning copy number via exogenous addition of an inducer (Supplementary Figure S2b). Further engineering, such as reducing the leaky expression level of RepL to decrease the baseline plasmid number could further improve tunability (34).

### Enhanced arginine production without a growth deficit using the synthetic decoy system

**Figure 4.**
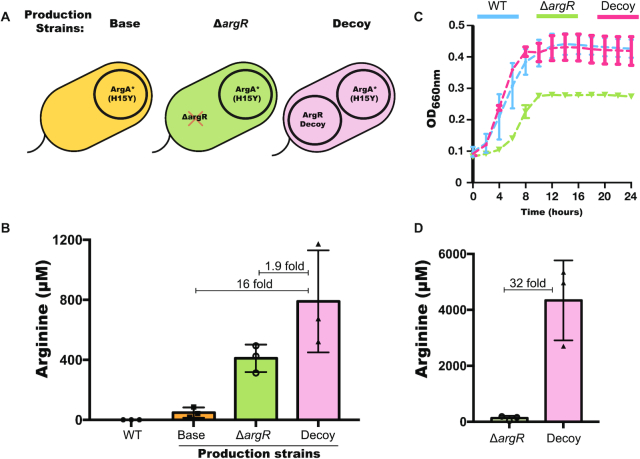
Titer and growth curves of arginine production strains. (**A**) Schematic view of the arginine production strains. (**B**) Arginine titer of production strains in 5 ml culture measured by LC–MS after 24 h. (**C**) Growth curves of different arginine production strains. Error bars show standard deviations from *n* = 3 biological replicates. (**D**) Arginine titer of production strains in 100 ml culture measured by LC–MS after 24 h.

To confirm the decoy effect and quantify its ultimate impact on arginine production, we introduced the ArgR decoy into an arginine production strain. This base production strain harbors a plasmid expressing a mutated version of *argA*, *argA* (H15Y), which we denote argA*, where allosteric feedback is removed (Figure 4A). To quantify arginine production, we used LC–MS to measure the titer of arginine in strains with and without the decoy after 24 h of fermentation. Since we observed significant up-regulation of arginine production pathway genes with our ArgR decoy system (Figure 2B), we reasoned that arginine production should also increase with the ArgR decoy. Indeed, we observed an average titer of 790 μM arginine after co-transforming ArgA* and the ArgR decoy system, a 16-fold increase in arginine titer compared to the same strain without the decoy (Figure 4B). These results confirm that the decoy system can effectively steer metabolic pathway activity, increasing production. Additionally, we observed a 1.9-fold increase in production of the decoy-based production strain compared to production from an *argR* knock-out strain (Figure 4B, Supplementary Table S2). Importantly, even with the increase in arginine production, we observed no detectable growth differences compared with the wide type strain (Figure 4C). This result is in contrast to strategies based on an *argR* knock-out, which have a significant growth deficit (Figure 4C). As culture volumes increase, we reasoned that the production discrepancy between the decoy and knock-out strain would be exacerbated due to the growth defect associated with the *argR* knock-out. Indeed, when scaling up the culture volume to 100 ml, which is smaller than the increase that would be needed for true production conditions, we observed a 32-fold difference between the decoy and knock-out strain (Figure 4D). These results suggest that the decoy system is an effective tool for redirecting metabolic flux that imposes a low burden to the cell and can lead to increased production compared to its knock-out counterpart.

### Increased genetic integrity of the production plasmid using the decoy system

Engineered cells that exhibit a growth deficit can eventually be out-competed by low-productivity counterparts that acquire a mutation that restores fitness. Because the production strain based on the *argR* deletion exhibits a significant growth deficit, we reasoned that using the knock-out strategy may select for mutations in the production plasmid, since mutations occurring in the ArgA* production plasmid could recover growth but decrease yields. However, since the decoy-based production strain shows negligible fitness differences compared to wild type, we anticipated that the selection for mutations would be lower.

**Figure 5.**
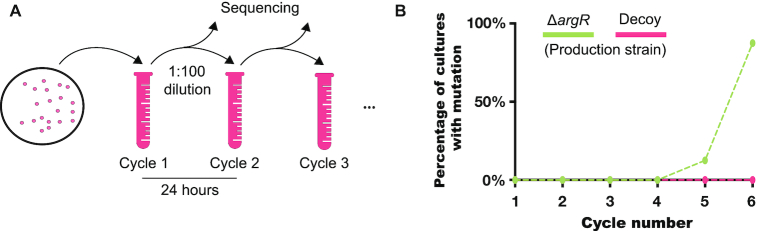
Genetic integrity of arginine production plasmids. (**A**) Schematic diagram of genetic integrity test. Eight different bacterial colonies from each production strain were subjected to continuous culture. Every 24 h, we isolated and sequenced regions of interest from the plasmids. The target sequencing regions of interest were argA* for the Δ*argR* production strain, and argA* and the decoy region in the decoy-based production strain. (**B**) Percentages show the number of bacterial cultures with a mutation out of *n* = 8 sequenced.

In the context of a bioprocess, the opportunity for a low productivity mutant to emerge and overtake the population increases as the culture is scaled up to large bioreactors because of the many divisions needed to reach adequate culture density. To mimic this, we sequentially diluted our production strains at a small scale over several days to maintain continuous growth. To assess the genetic integrity of *argR* deletion-based and decoy-based production strains, we quantified the mutations by sequencing the ArgA* plasmid and decoy plasmid at the end of each cycle (Figure 5A). In the knock-out production strain (Δ*argR*), we found that cells containing plasmids with mutations within the argA* coding sequence quickly took over the culture; seven out of eight colonies that we sequenced were mutated by cycle 6 (Figure 5B). Of these mutated plasmids, six of the seven contained Y15H, which reverts argA* to *argA* by restoring allosteric feedback (Supplementary Table S3). Growth curves at cycle 1 and cycle 6 show that growth is partially recovered in the knock-out strain following these mutations (Supplementary Figure S3). In contrast, the decoy-based production strain showed high genetic integrity, and the colonies that we sequenced contained no mutations in either argA* or the decoy region of the production plasmid or the decoy plasmid itself (Figure 5B).This result suggests that the decoy-based production strategy maintains higher genetic integrity than the burdensome *argR* deletion-based version, allowing for the maintenance of intact production from heterologous elements after many cycles of cell division.

### Improved pinene tolerance using a multiplexed decoy library

Since transcription factor decoys have the ability to perturb transcriptional programs and alter phenotypes, we reasoned that they could also enhance tolerance phenotypes. A library approach to tolerance screening is easily adaptable to decoy systems since the binding sequences are short and can be created by annealing oligos. Moreover, decoys do not rely on transcription or translation, making them amenable to high efficiency multi-part Golden Gate assemblies (37) without the need to ensure in-frame inserts. By matching oligo overhang sequences, or using palindromic overhang sequences, libraries of multiple decoy inserts can be created in streamlined one-pot reactions. As a showcase of decoy libraries, we sought to enhance tolerance to pinene, an important monoterpene that can be produced in *E. coli* (38). Pinene is of interest to metabolic engineers as it has many potential uses, such as an alternative jet fuel, flavoring and fragrance additive, and a therapeutic agent (38–40). Production of pinene is toxic to *E. coli* and growth is inhibited in 0.5% pinene (v/v) (Supplementary Figure S4). However, several studies have demonstrated that *E. coli* can cope with pinene-induced stress using endogenous genes (41–43).

**Figure 6.**
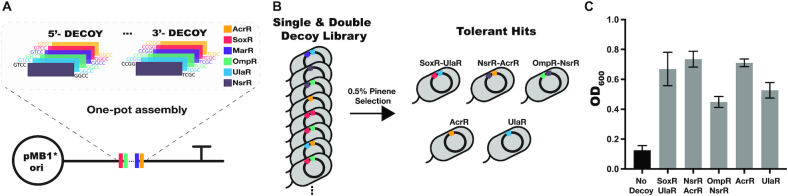
Multiplexed decoy library for pinene tolerance. (**A**) Cloning scheme to create combinatorial decoy libraries. The transcription factor binding site sequences are on oligos that are annealed, followed by a single-pot Golden Gate assembly. In the schematic, the double decoy design is shown. (**B**) A pooled library of single and double decoy combinations were grown in pinene to select for decoys that increase tolerance. Three decoy pairs (SoxR-UlaR, NsrR-AcrR, OmpR-NsrR) and two single decoys (AcrR, UlaR) were identified from the selection. (**c**) Optical density at 600 nm (OD_600_) of hits grown in 0.5% pinene (v/v) for 24 h. Error bars show standard deviations from *n* = 4 biological replicates.

To test whether we could rapidly screen for improved pinene tolerance using a multiplexed library approach, we created a decoy library on a high copy plasmid. The library is based on regulators of genes known to play a role in pinene tolerance and contains all single and double decoy combinations (Supplementary Table S4). Single and double decoy libraries were constructed in pooled, single-pot reactions, highlighting the simplicity of the decoy-based design (Figure 6A). The pooled reactions were transformed and a small subset of the transformations were plated and individual colonies sequenced to ensure adequate coverage. We then subjected the pooled library to 0.5% pinene selection to identify tolerant variants (Figure 6B). We performed six parallel selection experiments. In one of the experiments, we did not recover growth after 48 h. In the other five cases, the selection experiments all converged such that all cells contained a single plasmid. In these experiments we found the following single and double decoys: SoxR-UlaR, NsrR-AcrR, OmpR-NsrR, AcrR and UlaR. To validate each decoy and rule out genomic mutations as the cause of tolerance, the decoy plasmid was isolated and re-transformed into fresh cells. These cells were compared to cells containing the same plasmid lacking the decoy, which served as a negative control. Indeed, each decoy returned from the pinene selection experiment exhibited enhanced tolerance relative to the negative control as measured by OD_600_ at 24 h in 0.5% pinene (Figure 6C).

For each of the double decoy hits, we constructed single decoy versions to evaluate whether the combination of binding sites was critical for improved tolerance (Supplementary Figure S5). In the case of SoxR-UlaR, the UlaR site is sufficient for tolerance, as this hit was returned in the original selection. Similarly, for the NsrR-AcrR decoy, the initial screen demonstrated that the AcrR decoy alone can increase tolerance. The other possible single decoys (SoxR, NsrR and OmpR) resulted in highly variable growth in pinene (Supplementary Figure S5). Tolerance was not enhanced consistently across biological replicates, suggesting that in certain cases decoy combinations may be necessary for robust growth in pinene. Similarly, when we decreased the copy number of the SoxR-UlaR decoy by switching to a p15a origin of replication (∼10 copies per cell), from a pMB1* origin of replication (∼500 copies), tolerant phenotypes were highly variable (Supplementary Figure S5), suggesting that the number of decoy sites is critical for the increase in tolerance. Taken together, these results demonstrate the straightforward application of multiplexed decoy-based approaches to library selection.

## DISCUSSION

We have harnessed decoy binding sites to titrate transcription factors in order to regulate expression of genes with minimal impact on fitness, therefore increasing production in a metabolic engineering context. We have shown that transcription factor decoys are an effective tool for altering gene expression for both native and heterologous targets. Importantly, the effect of the decoy can be tuned by changing its copy number or DNA sequence. As an application, we used a decoy system to control arginine biosynthesis and showed that it can regulate metabolic flux by increasing transcriptional activity of the arginine production pathway, resulting in a 16-fold increase in arginine production compared to a parental strain lacking the decoy. In contrast to production strains based on an *argR* knock-out, the decoy system exhibits no detectable growth difference compared to wild type while producing more arginine. This suggests that using the decoy to selectively titrate away transcription factors may have a much smaller burden compared to alternative strategies. Since fitness deficits can select for low-producing mutants, we also compared the number of mutations between alternative designs in the key arginine synthesis enzyme ArgA*. We found that the production strain based on the decoy system maintains genetic integrity of the production plasmid while the knock-out system does not. Further, by screening for pinene tolerance, we have shown that the method is highly amenable to multiplexing. It is feasible to scale up library diversity by increasing the number of decoy inserts by using palindromic overhangs or increasing the number of decoys in the library.

In comparison to alternative gene regulation tools, such as regulatory RNAs or CRISPR-based regulation, transcription factor decoys require fewer cellular resources since the effect does not rely on transcription or translation. Furthermore, the absence of transcription or translation reduces the opportunity for off-target effects from overexpression, which have been associated with dCas9 in bacteria (19,20). We speculate that these factors contribute to the relative fitness advantage of an ArgR decoy over CRISPRi approaches. Antisense RNA, another strategy for programmable gene regulation, can be sensitive to RNA stability for effective silencing (44), potentially limiting utility. However, we note that for contexts where regulation is affected both positively and negatively by transcription factors, the use of transcription factor decoys may not be straightforward, and it may be necessary to adopt other regulatory strategies.

The design simplicity adds to the appeal of decoy based transcriptional regulation. For example, decoy systems could be used for cases in which the exact transcription factor binding site is unknown simply by using the putative promoter region as the decoy. Furthermore, this approach can extend to organisms beyond *E. coli* where tunable expression systems and synthetic biology tools are more limited. Prokaryotic systems that rely heavily on negative regulation are likely to be amenable to regulation using transcription factor decoys. In metabolic engineering, this is of particular interest for non-model organisms that have the ability to grow on desired feedstocks, such as cellulosic biomass or even through photosynthesis. In these non-model organisms, decoys could potentially be applied to steer biosynthesis towards desired end products.

## Supplementary Material

gkaa1234_Supplemental_File
