# Supplementary material for: Programmable gene regulation for metabolic engineering using decoy transcription factor binding sites
Source: Nucleic Acids Res. 2020 Dec 24;49(2):1163–72. doi: 10.1093/nar/gkaa1234 (PMC7826281; doi:10.1093/nar/gkaa1234)
Supplement: gkaa1234_Supplemental_File [file gkaa1234_supplemental_file.docx]

**SUPPLEMENTARY INFORMATION**

**Figure S1. Decoy activation of P_lac_-RFP over time.** Time course of expression levels for data shown in Fig. 1b. IPTG induction (1 mM) for ‘fully induced’ trial starts at time t = 0. Error bars show standard error from n = 3 biological replicates.


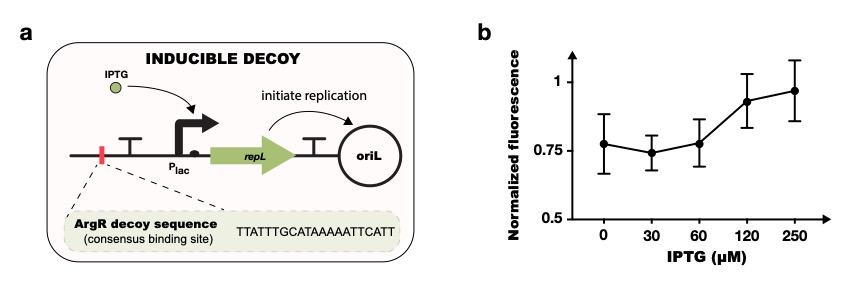


**Figure S2. Tunable control of the ArgR decoy system with inducible copy number plasmid.** **(a)** Schematic view of the design of the inducible ArgR decoy system. **(b)** ArgR decoy effect is enhanced as IPTG increases the plasmid copy number (0.6 mM arginine treatment). Note that the y-axis starts at 0.5. Error bars show standard error from n = 3 biological replicates.

**Figure S3. Growth curves of production strains before and after growth cycles.** Cultures correspond to data in Fig. 5b taken at cycle 1 and 6 for **(a)** Δ*argR* production strain and **(b)** ArgR decoy production strain. Error bars show standard error from n = 8 biological replicates.

**Figure S4. Pinene tolerance of wild type *E. coli* BW25113.** Endpoint OD_600_ of cultures grown with α-pinene. Error bars show standard error from n = 3 biological replicates.

**Figure S5. Pinene tolerance of decoy variants.** Variants of hits from Fig. 6c grown in 0.5% α-pinene (v/v). All single decoy variants were tested on the same high copy backbone as the double decoys (pMB1*). In addition, we tested the SoxR-UlaR decoy on a ‘low copy’ backbone by using the p15A origin of replication. Error bars show standard error for n ≥ 4 biological replicates.

**Table S1. Sequences from ArgR decoy library in Fig. 3b**

| Rank | Sequence |
| --- | --- |
| 1 | ATTTTTGCATAATTATTCATT |
| 2 | ATAATTGCATGATTATTCATT |
| 3 | ATTTATGCATAAAAATTCAGT |
| 4 | TTAATTGAATAATTATTCATT |
| 5 | TTAAATGAATAAATATTCATT |
| 6 | TTTATTGAATGAAAATTCATT |
| 7 | TTTAATGCATGAATATTCATT |
| 8 | TTTTATGAATAATAATTCAGT |
| 9 | TTTTTTGCATGATAATTCATT |
| 10 | TTTTTTGAATAAATATTCATT |
| 11 | ATTTATGCATGATTATTCACT |
| 12 | TTATTTGAATAATAATTCACT |
| 13 | TTTTTTGAATGATAATTCATT |
| 14 | ATATTTGAATGATAATTCATT |
| 15 | ATTTATGAATAAATATTCATT |
| 16 | TTATATGCATAAATATTCAGT |
| 17 | ATATATGAATAATAATTCATT |
| 18 | TTATTTGCATGAATATTCACT |
| 19 | TTAAATGCATAAAAATTCAGT |
| 20 | TTTATTGCATAATTATTCAGT |
| 21 | TTTAATGCATGAATATTCTTT |
| 22 | TATATTGCATGAAAATTCATT |
| 23 | TAAAATGCATGATAATTCACT |
| 24 | TAATTTGAATGATTATTCAGT |
| 25 | AATATTGAATGAATATTCAGT |
| 26 | TATTTTGCATAAAAATTCAGT |
| 27 | TATATTGCATGAATATTCATT |
| 28 | ATTTTTGAATGAAAATTCACT |

**Table S2. Productivities of decoy and Δ*argR* production strains**

Mean productivity at two time points of a 5 mL

culture ± standard error from n = 3 biological replicates.

|  | **Productivity (µmol/L.hr)** | |  |
| --- | --- | --- | --- |
| **Strain** | | **6 hours** | **24 hours** |
| **ArgR Decoy + ArgA*** | | 13.4 ± 5.6 | 32.9 ± 8.2 |
| **Δ*argR* + ArgA*** | | 7.1 ± 2.2 | 17.1 ± 2.2 |

**Table S3. Mutations found in ArgA* in the strain ArgA*/Δ*argR* at cycle 6**

| **Colony #** | **Mutation** |
| --- | --- |
| 1 | *A197R* |
| 2 | None |
| 3 | *Y15H* |
| 4 | *Y15H* |
| 5 | *Y15H* |
| 6 | *Y15H* |
| 7 | *Y15H, T93H* |
| 8 | *Y15H* |

**Table S4. Pinene tolerance library decoy sequences**

| **Decoy** | **Forward Sequence** |
| --- | --- |
| AcrR | GATTTACATACATTTNTGAATGTATGTA |
| SoxR | GAACCCTCAAGTTAACTTGAGG |
| MarR | ACTAATTACTTGCCAGGGCAAGTAAT |
| OmpR | TTTACTTTTGGTTACATCTA |
| UlaR | TGATTAATCATGAACAATCA |
| NsrR | GATGCATTTAAAATACATC |

**Sequence: ArgR Consensus Decoy Plasmid**

Decoy plasmid used for arginine production based on pBbE5a-RFP from the BglBrick vector collection (1). The decoy sequence is shown in bold.

gacgtcggtgcctaatgagtgagctaacttacattaattgcgttgcgctcactgcccgctttccagtcgggaaacctgtcgtgccagctgcattaatgaatcggccaacgcgcggggagaggcggtttgcgtattgggcgccagggtggtttttcttttcaccagtgagacgggcaacagctgattgcccttcaccgcctggccctgagagagttgcagcaagcggtccacgctggtttgccccagcaggcgaaaatcctgtttgatggtggttaacggcgggatataacatgagctgtcttcggtatcgtcgtatcccactaccgagatgtccgcaccaacgcgcagcccggactcggtaatggcgcgcattgcgcccagcgccatctgatcgttggcaaccagcatcgcagtgggaacgatgccctcattcagcatttgcatggtttgttgaaaaccggacatggcactccagtcgccttcccgttccgctatcggctgaatttgattgcgagtgagatatttatgccagccagccagacgcagacgcgccgagacagaacttaatgggcccgctaacagcgcgatttgctggtgacccaatgcgaccagatgctccacgcccagtcgcgtaccgtcttcatgggagaaaataatactgttgatgggtgtctggtcagagacatcaagaaataacgccggaacattagtgcaggcagcttccacagcaatggcatcctggtcatccagcggatagttaatgatcagcccactgacgcgttgcgcgagaagattgtgcaccgccgctttacaggcttcgacgccgcttcgttctaccatcgacaccaccacgctggcacccagttgatcggcgcgagatttaatcgccgcgacaatttgcgacggcgcgtgcagggccagactggaggtggcaacgccaatcagcaacgactgtttgcccgccagttgttgtgccacgcggttgggaatgtaattcagctccgccatcgccgcttccactttttcccgcgttttcgcagaaacgtggctggcctggttcaccacgcgggaaacggtctgataagagacaccggcatactctgcgacatcgtataacgttactggtttcacattcaccaccctgaattgactctcttccgggcgctatcatgccataccgcgaaaggttttgcgccattcgatggtgtccgggatctcgacgctctcccttatgcgactcctatggccctgtcctgctgccaatggcgagtagcgaagacgttatcaaagagttcatgcgtttcaaagttcgtatggaaggttccgttaacggtcacgagttcgaaatcgaaggtgaaggtgaaggtcgtccgtacgaaggtacccagaccgctaaactgaaagttaccaaaggtggtccgctgccgttcgcttgggacatcctgtccccgcagttccagtacggttccaaagcttacgttaaacacccggctgacatcccggactacctgaaactgtccttcccggaaggtttcaaatgggaacgtgttatgaacttcgaagacggtggtgttgttaccgttacccaggactcctccctgcaagacggtgagttcatctacaaagttaaactgcgtggtaccaacttcccgtccgacggtccggttatgcagaaaaaaaccatgggttgggaagcttccaccgaacgtatgtacccggaagacggtgctctgaaaggtgaaatcaaaatgcgtctgaaactgaaagacggtgggggctgactg**ttatttgcataaaaattcatt**tgtatgcacagctgaaggtcgtcactccaccggtgcttaaggatccaaactcgagtaaggatctccaggcatcaaataaaacgaaaggctcagtcgaaagactgggcctttcgttttatctgttgtttgtcggtgaacgctctctactagagtcacactggctcaccttcgggtgggcctttctgcgtttatacctagggcgttcggctgcggcgagcggtatcagctcactcaaaggcggtaatacggttatccacagaatcaggggataacgcaggaaagaacatgtgagcaaaaggccagcaaaaggccaggaaccgtaaaaaggccgcgttgctggcgtttttccataggctccgcccccctgacgagcatcacaaaaatcgacgctcaagtcagaggtggcgaaacccgacaggactataaagataccaggcgtttccccctggaagctccctcgtgcgctctcctgttccgaccctgccgcttaccggatacctgtccgcctttctcccttcgggaagcgtggcgctttctcatagctcacgctgtaggtatctcagttcggtgtaggtcgttcgctccaagctgggctgtgtgcacgaaccccccgttcagcccgaccgctgcgccttatccggtaactatcgtcttgagtccaacccggtaagacacgacttatcgccactggcagcagccactggtaacaggattagcagagcgaggtatgtaggcggtgctacagagttcttgaagtggtggcctaactacggctacactagaaggacagtatttggtatctgcgctctgctgaagccagttaccttcggaaaaagagttggtagctcttgatccggcaaacaaaccaccgctggtagcggtggtttttttgtttgcaagcagcagattacgcgcagaaaaaaaggatctcaagaagatcctttgatcttttctacggggtctgacgctcagtggaacgaaaactcacgttaagggattttggtcatgactagtgcttggattctcaccaataaaaaacgcccggcggcaaccgagcgttctgaacaaatccagatggagttctgaggtcattactggatctatcaacaggagtccaagcgagctcgtaaacttggtctgacagttaccaatgcttaatcagtgaggcacctatctcagcgatctgtctatttcgttcatccatagttgcctgactccccgtcgtgtagataactacgatacgggagggcttaccatctggccccagtgctgcaatgataccgcgagacccacgctcaccggctccagatttatcagcaataaaccagccagccggaagggccgagcgcagaagtggtcctgcaactttatccgcctccatccagtctattaattgttgccgggaagctagagtaagtagttcgccagttaatagtttgcgcaacgttgttgccattgctacaggcatcgtggtgtcacgctcgtcgtttggtatggcttcattcagctccggttcccaacgatcaaggcgagttacatgatcccccatgttgtgcaaaaaagcggttagctccttcggtcctccgatcgttgtcagaagtaagttggccgcagtgttatcactcatggttatggcagcactgcataattctcttactgtcatgccatccgtaagatgcttttctgtgactggtgagtactcaaccaagtcattctgagaatagtgtatgcggcgaccgagttgctcttgcccggcgtcaatacgggataataccgcgccacatagcagaactttaaaagtgctcatcattggaaaacgttcttcggggcgaaaactctcaaggatcttaccgctgttgagatccagttcgatgtaacccactcgtgcacccaactgatcttcagcatcttttactttcaccagcgtttctgggtgagcaaaaacaggaaggcaaaatgccgcaaaaaagggaataagggcgacacggaaatgttgaatactcatactcttcctttttcaatattattgaagcatttatcagggttattgtctcatgagcggatacatatttgaatgtatttagaaaaataaacaaataggggttccgcgcacatttccccgaaaagtgccacct

**References**

1. Lee,T.S., Krupa,R.A., Zhang,F., Hajimorad,M., Holtz,W.J., Prasad,N., Lee,S.K. and Keasling,J.D. (2011) BglBrick vectors and datasheets: A synthetic biology platform for gene expression. *J. Biol. Eng.*, **5**, 12.
